# Herb and Flowers of *Achillea millefolium* subsp. *millefolium* L.: Structure and Histochemistry of Secretory Tissues and Phytochemistry of Essential Oils

**DOI:** 10.3390/molecules28237791

**Published:** 2023-11-27

**Authors:** Agata Konarska, Elżbieta Weryszko-Chmielewska, Aneta Sulborska-Różycka, Anna Kiełtyka-Dadasiewicz, Marta Dmitruk, Małgorzata Gorzel

**Affiliations:** 1 Department of Botany and Plant Physiology, University of Life Sciences in Lublin, Akademicka 15, 20-950 Lublin, Poland; agata.konarska@up.lublin.pl (A.K.); elaweryszko@wp.pl (E.W.-C.); marta.dmitruk@up.lublin.pl (M.D.); 2 Department of Plant Production Technology and Commodities Science, University of Life Sciences in Lublin, 20-950 Lublin, Poland; anna.kieltyka-dadasiewicz@up.lublin.pl; 3 Garden of Cosmetic Plants and Raw Materials, Research and Science Innovation Center, 20-819 Lublin, Poland; seminariumgorzel@wp.pl; 4 Faculty of Health Sciences, Vincent Pol University in Lublin, 20-816 Lublin, Poland

**Keywords:** Asteraceae, yarrow, glandular trichomes, secretory canals, secretion compounds

## Abstract

*Achillea millefolium* L. herb and flowers have high biological activity; hence, they are used in medicine and cosmetics. The aim of this study was to perform morpho-anatomical analyses of the raw material, including secretory tissues, histochemical assays of the location of lipophilic compounds, and quantitative and qualitative analysis of essential oil (EO). Light and scanning electron microscopy techniques were used to analyse plant structures. The qualitative analyses of EO were carried out using gas chromatography-mass spectrometry (GC/MS). The results of this study showed the presence of exogenous secretory structures in the raw material, i.e., conical cells (papillae) on the adaxial surface of petal teeth and biseriate glandular trichomes on the surface flowers, bracts, stems, and leaves. Canal-shaped endogenous secretory tissue was observed in the stems and leaves. The histochemical assays revealed the presence of total, acidic, and neutral lipids as well as EO in the glandular trichome cells. Additionally, papillae located at the petal teeth contained neutral lipids. Sesquiterpenes were detected in the glandular trichomes and petal epidermis cells. The secretory canals in the stems were found to contain total and neutral lipids. The phytochemical assays demonstrated that the *A. millefolium* subsp. *millefolium* flowers contained over 2.5-fold higher amounts of EO (6.1 mL/kg) than the herb (2.4 mL/kg). The EO extracted from the flowers and herb had a similar dominant compounds: β-pinene, bornyl acetate, (E)-nerolidol, 1,8-cineole, borneol, sabinene, camphor, and α-pinene. Both EO samples had greater amounts of monoterpenes than sesquiterpenes. Higher amounts of oxygenated monoterpenes and oxygenated sesquiterpenoids were detected in the EO from the herb than from the flowers.

## 1. Introduction

The genus *Achillea* L. from the family Asteraceae comprises approximately 110–140 species [1], many of which are used in the food, pharmaceutical, and cosmetic industries. The representatives of the genus have a long history of use in traditional medicine around the world [2,3,4,5]. One of the oldest known botanicals used by humans is *Achillea millefolium* L. (yarrow), a cosmopolitan perennial growing in the temperate climate of Eurasia, America Northern, and Australia [6,7]. *Achillea millefolium* represents a highly polymorphic group of closely related species, subspecies, microspecies, and hybrids, which differ in the ploidy level, morphology, and chemical composition [8,9,10]. There are five most common subspecies of *A. millefolium*: subsp. *millefolium* L., subsp. *alpestris* (Wimm. & Grab.) Gremli, subsp. *ceretanum* Sennen, subsp. *borealis* (Bong.) Breitung, and subsp. *sudetica* (Opiz) Oborny [6,11].

In Poland, *A. millefolium* is most often found in meadows, roadsides, slopes, field margins, and wastelands, but it can also be cultivated as an ornamental and spice plant [12]. The plants have shoots with abundant trichomes, tri- or tetrapinnate leaves, and an intense aroma. *A. millefolium* blooms from June to October or even until the first frost.

In many European and Asian countries, *A. millefolium* is used in folk and conventional medicine as well as cosmetology [13,14,15,16]. The medicinal and cosmetic raw material of yarrow is its herb (*Millefolii herba*) and flowers (*Millefolii flos*) collected from freshly blooming plants only in sunny areas [7,17,18]. Extracts from yarrow herb and flowers contain valuable biologically active compounds, with the characteristic blue or blue-green EO as the most important substance [19,20]. It is rich in azulenes, e.g., chamazulene [21] and such sesquiterpenes as leucodin and achillin [22,23]. Azulenes, which are produced from proazulenes during steam distillation processes, are of great importance in medicine mainly due to their anti-allergy, anti-inflammatory, anticancer, antidiabetic, antimicrobial, and antifungal properties [24]. Yarrow raw material also contains phenolic acids [25,26], flavonoids [27,28], saponins [29], and phytosterols [30].

In folk and modern medicine, alcoholic and aqueous extracts from herb and flowers as well as *A. millefolium* EO are used for the treatment of skin disorders in renal injury [31] and memory disorders [32], for a reduction of the intensity and duration of menstrual bleeding [33], and in many other diseases [21,34,35,36]. Previous studies have shown that yarrow is generally safe and well tolerated by the human organism [37]; however, when used in excess, it may cause headaches and/or stupor [38] and trigger allergic contact dermatitis [39].

Yarrow raw materials have numerous applications in cosmetics [15,16]. Extracts and EO are added to soothing and regenerating masks, facial creams, lotions, shampoos, and toothpastes, whereas dried herb is applied externally as part of hair and skin care and to prepare relaxing baths [16,40]. They are characterised by multifunctional properties when used as active ingredients of cosmetics, i.e., they serve cleansing, moisturising, soothing, conditioning, masking, refreshing, and odorising functions [37,41]. They can be used for the production of innovative cosmetics designed to protect the skin from the adverse impact of air pollutants and climatic factors [42]. Yarrow EO is also used in the treatment of skin inflammation or as protection from sunburns [15,43].

The bitter-salty and spicy young leaves of the plant can be used as a seasoning for sauces, soups, and fish [44,45]. Yarrow inflorescences are used to prepare teas [46], while yarrow EO is added to liqueurs and tinctures [12]. Additionally, the active substances contained in these plants have repellent and insecticidal properties [47].

The stems, leaves, flowers, and fruits of many *Achillea* species, including *A. millefolium*, are equipped with glandular and non-glandular trichomes [48,49,50,51] and secretory canals [50,52]. These exo- and endogenous secretory structures are regarded as the main sites of accumulation of bioactive substances in the organs of medicinal and cosmetic plants from the family Asteraceae [53,54,55].

While there is relatively vast knowledge of the phytochemistry, biological activity, and applications of *A. millefolium* plants, the information on the structure of secretory tissues in this species and the location of bioactive substances is incomplete. The literature data also indicate that the content of bioactive compounds in yarrow raw material depends on the region of origin of the plant [56,57,58,59,60]. Therefore, the aim of the current study was (i) to present the structure of secretory tissues of *Achillea millefolium* subsp. *millefolium* flowers, stems, and leaves for better anatomical diagnostics of raw material, (ii) to compare the location of EO contained in different parts of shoots using histochemical assays for better characterisation of secretory tissues, and (iii) to determine the quantitative and qualitative composition of EO in yarrow herb and flowers from south-eastern Poland with the use of phytochemical analyses.

## 2. Results

### 2.1. Secretory Structures of Flowers and Bracts

**Figure 1:**
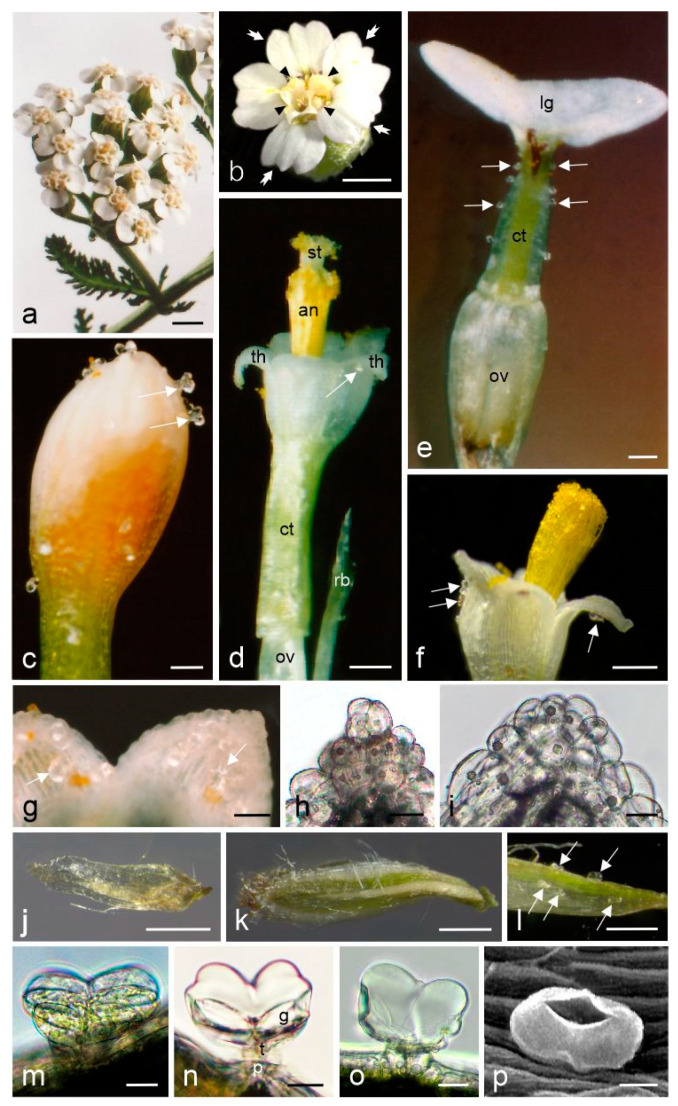
Morphology of *Achillea millefolium* subsp. *millefolium* inflorescences and flowers; (**a**–**g**,**j**–**l**)—stereoscopic microscopy (SM), (**h**,**i**,**m**–**o**)—light microscopy (LM), (**p**)—scanning electron microscopy (SEM). (**a**) Fragment of the upper stem part with capitula in the full-bloom stage; (**b**) General view of a capitula; white arrowheads indicate ray flowers, black arrowheads indicate disc flowers; (**c**) Fragment of the upper part of a corolla of a disc flower bud with visible glandular trichomes (white arrows); (**d**) Fragment of a disc flower in the full-bloom stage (pistil stage); white arrow indicates a glandular trichome; (**e**) Fragment of a ray flower with glandular trichomes (white arrows) on the corolla tube; (**f**) Fragment of the upper part of a disc flower (stamen stage) with visible glandular trichomes on the adaxial surface of corolla teeth (white arrows); (**g**) Adaxial surface of corolla teeth with glandular trichomes (white arrows); (**h**,**i**) Conical cells (papillae) on the top of corolla teeth; (**j**) Receptacular bract; (**k**,**l**) Involuclar bracts with glandular trichomes (white arrows); (**m**–**p**) Glandular trichomes on the adaxial surface of the corolla of disc flowers; st—style, an—anthers, th—corolla teeth, ct—corolla tube, lg—ligule, ov—ovary, rb—receptacular bract; g—glandular cell; t—stalk cell; p—basal cell. Scale bars: 1 mm (**a**), 500 μm (**b**), 200 µm (**j**,**k**); 100 µm (**d**–**f**,**l**), 50 µm (**c**), 25 µm (**g**–**i**), 20 µm (**m**–**p**).

The inflorescences of *A. millefolium* subsp. *millefolium* formed dense corymbose clusters at shoot apices (Figure 1a) and consisted of small capitula (Figure 1b). In the peripheral part of the capitula, there were sparse (5) and most often white ray flowers, while the central part of the inflorescence was occupied by disc flowers (8–18) characterised by a whitish 5-toothed corolla (Figure 1b).

A characteristic trait of the epidermis of the ray and disc flower petals was the presence of conical cells (papillae) on the adaxial surface of the teeth (Figure 1h,i). Biseriate glandular trichomes were present on the surface of the petals in both the disc flowers (Figure 1c,d,f,g) and the ray flowers (Figure 1e). Most of the glandular trichomes in the disc flowers were located in the upper part of the perianth and were clearly visible on flower buds (Figure 1c) and on corolla teeth in opened flowers (Figure 1f,g). The trichomes in the ray flowers were mainly located in the tubular lower part of the corolla (Figure 1e). They were occasionally present on the ovary but absent in the ligulate upper part of the corolla. Biseriate glandular trichomes were also observed on the surface of the receptacular bracts and involuclar bracts (Figure 1j,k), with the greatest density on their abaxial surface (Figure 1l).

The biseriate glandular trichomes with a height of 56.5–82.0 µm and a width of 77.0–108.0 µm (Table 1) observed in the flowers of *A. millefolium* subsp. *millefolium* were composed of 4–5 tiers of cells, including basal cells, stalk cells, and glandular cells constituting the head (Figure 1m–p). The cuticle on the surface of glandular cells detached from the cellulose walls during the secretion phase, thus forming a vesicle with accumulating secretion (Figure 1n,o). When the secretion filled the subcuticular space, the cuticle burst and released its contents (Figure 1p).

### 2.2. Non-Secretory and Secretory Structures of Stems and Leaves

**Figure 2:**
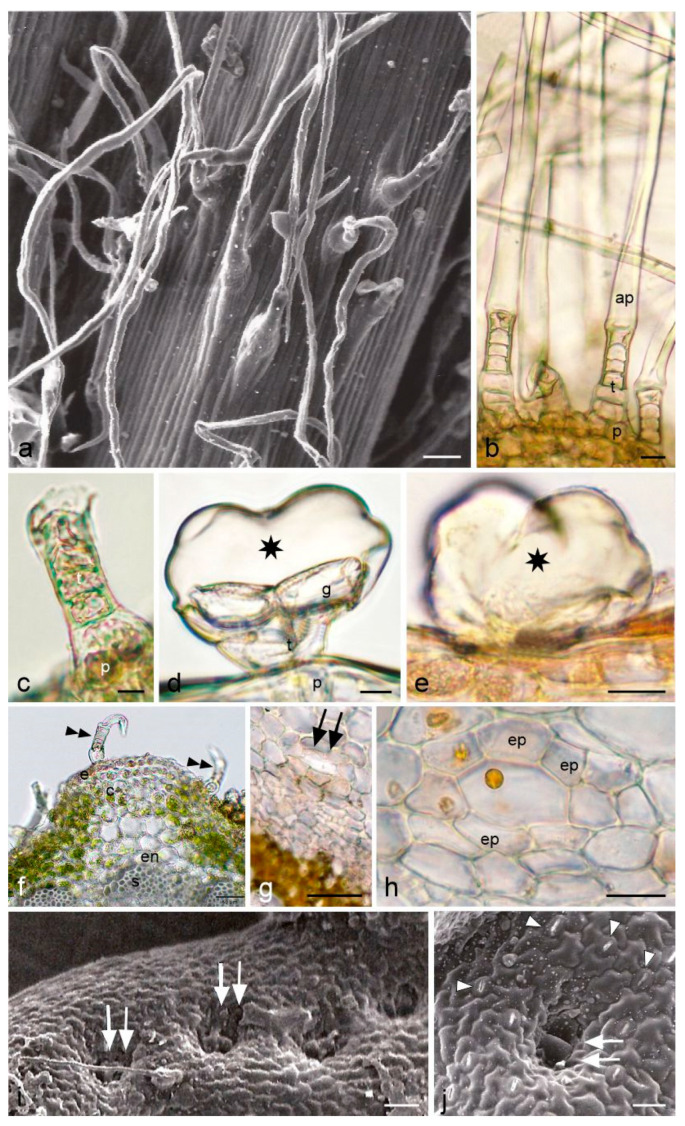
Non-secretory and secretory structures of *Achillea millefolium* subsp. *millefolium* stems and leaves. (**a**,**i**,**j**)—SEM, (**b**–**h**)—LM. (**a**) Non-glandular trichomes visible on the surface of the stem; (**b**) Fragment of the stem with uniseriate non-glandular trichomes with a strongly elongated apical cell; (**c**) Fragment of a non-glandular trichome on the stem surface without an elongated apical cell; (**d**,**e**) Glandular trichomes on the abaxial surface of the leaf (**d**) and the stem (**e**); stars indicate the subcuticular space; (**f**) Fragment of a cross-section of the stem with non-glandular trichomes (double black arrowheads); (**g**,**h**) Secretory canals (double black arrows—**g**) visible on the cross-section of the stem; (**i**,**j**)—cavities with secretory cells (white double arrows) on the adaxial surface of leaves; (**j**) white arrowheads indicate stomata; p—trichome basal cell, g—glandular cell; t—stalk cell, ap—apical cell, ep—epithelial cell, e—epidermis, c—collenchyma, en—endodermis, s—sclerenchyma. Scale bars: 50 μm (**a**,**f**,**g**), 20 µm (**b**,**e**,**h**–**j**), 10 µm (**c**,**d**).

The stems and leaves of *A. millefolium* subsp. *millefolium* were found to bear numerous non-glandular and biseriate glandular trichomes. The long non-glandular trichomes present on these organs formed a greyish web-like coating (Figure 2a). The length of non-glandular trichomes was in a wide range of 59–2800 µm, depending on the organ (Table 2). These trichomes were uniseriate and consisted of a basal cell, 4–5 stalk cells, and a highly elongated whip-like apical cell (Figure 2a,b). Due to the fragility of these trichomes, it was possible to observe only basal and stalk cells with viable content in the microscopic preparations (Figure 2c,f). In turn, the apical cell of the non-glandular trichomes was initially viable but its protoplast gradually died.

The glandular trichomes on the yarrow stems and leaves had a similar structure to that of the floral trichomes (Figure 2d), but they were shorter and had a narrower head (Table 2), and were also less numerous. They were often located in small cavities (Figure 2e).

Circular or oval secretory canals were observed in the cross-sections of the stems and leaves. The canals were formed through schizogeny, and their lumen enlarged as the cells gradually moved apart. Each canal in the stems and leaves was surrounded by one layer of epithelium composed of 4–6 cells (Figure 2g,h). Secretion droplets were often observed inside the canals (Figure 2h). The diameter of the secretory canals in the stems was in the range of 24.5–42.7 µm (average 34.4 µm). The diameter of the canals in the leaves ranged from 30.8 to 64.3 µm (average 45.2 µm).

The canals in the stems and leaf blade were most often located in the vicinity of phloem-xylem bundles. In the stems, there were two canals on the sides of each vascular bundle (one on each side) in the endodermis region, and sometimes additional canals were found outside the bundle. In turn, the canals in the leaves were located laterally to larger vascular bundles immediately at the xylem or at the phloem-xylem boundary and, less often, laterally to the xylem. A single bundle was accompanied by 1–2 cisterns; however, they were not observed in every bundle. The cross-sections of the stems revealed the presence of 14–15 canals, whereas 4–5 canals were observed in the cross-sections of the leaves.

The scanning electron microscope observations showed the presence of cavities in the adaxial part of the yarrow leaf blade. In the top view, these structures had a nearly round shape and were arranged in series along individual sections of the pinnate leaf or at its base. The cavity was divided into two parts by a visible wall, and the surface of the surrounding epidermis exhibited the presence of clusters of solidified secretion (Figure 2i,j).

### 2.3. Histochemical Analysis

**Figure 3:**
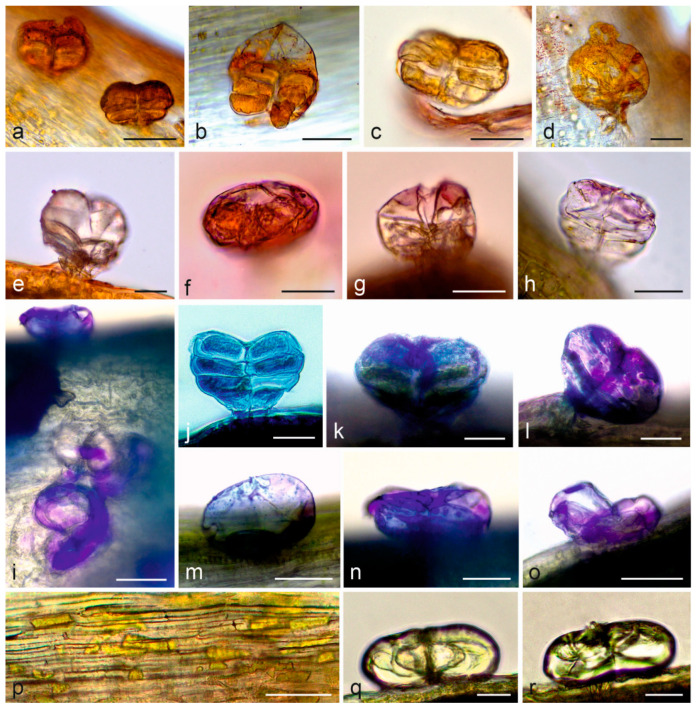
Histochemistry of *Achillea millefolium* subsp. *millefolium* disc flowers; (**a**–**o**,**q**,**r**) Biseriate glandular trichomes on the adaxial surface of corolla; (**a**–**e**) tests with Sudan IV identifying EO (orange color), (**f**–**h**) tests with Neutral red identifying EO (red color); (**i**–**o**) tests with Nile blue identifying acidic (blue color) and neutral (violet color) lipids; (**p**) Sesquiterpenes (yellow color) present in the cells of disc flower epidermis; (**q**,**r**) Glandular trichomes with secretion indicating the presence of sesquiterpenes—yellow color (test with concentrated sulphuric acids). Scale bars: 50 µm (**a**,**i**,**p**), 30 µm (**b**–**h**,**j**–**o**,**q**,**r**).

Flower: In the control preparations, the secretion contained in the glandular trichomes had a pale yellow-green color. EO and other lipophilic compounds, i.e., total, acidic, and neutral lipids, as well as sesquiterpenes, were detected in the biseriate glandular trichomes present on the surface of the corolla teeth and tubes in the disc flowers (Table 3; Figure 3a–o,q,r). Sesquiterpenes were also contained in the epidermis cells of corolla teeth and tubes (Figure 3p), while neutral lipids were additionally detected in papillae present at the apex of the corolla teeth and in the glandular trichomes of the receptacular bracts. The glandular trichomes exhibited various stages of development and different metabolic activity. In the pre-secretory stage, EO filled the trichome cells abundantly (Figure 3a–d,f), but their amount decreased substantially after the secretion process, as indicated by the lower intensity of staining of the trichome cell content, the empty subcuticular spaces, and the frequently observed cuticle rupture (Figure 3e,g,h). In the pre-secretory stage, acidic lipids were accumulated first and dominated in the trichome cells (Figure 3j,k). In turn, the content of neutral lipids present in the trichomes in the secretory and post-secretory stages increased (Figure 3i,l–o).

**Figure 4:**
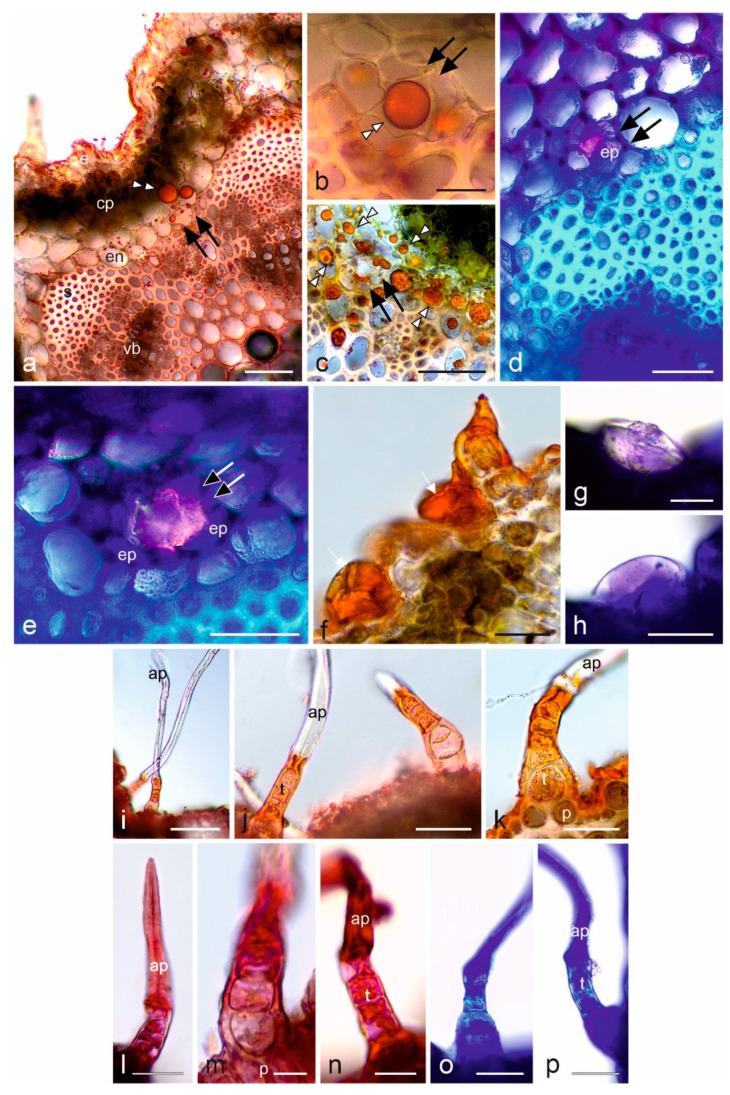
Histochemistry of *Achillea millefolium* subsp. *millefolium* stem; (**a**–**c**,**f**,**i**–**k**) tests with Sudan IV, (**d**,**e**,**g**,**h**,**o**,**p**) tests with Nile blue, (**l**–**n**) tests with Neutral red. (**a**–**c**) Fragments of cross-sections with secretory canals (double black arrows) and droplets of EO (white double arrowheads); (**d**,**e**) Secretory canals (double black arrows) with pink secretion in cross-sections; (**f**) EO present in glandular trichomes (white arrows); (**g**,**h**) Neutral lipids present in glandular trichomes; (**i**–**p**) Non-glandular trichomes with cells containing lipophilic compounds—total (**i**–**n**) and acidic (**o**,**p**) lipids; e—epidermis, cp—cortex parenchyma, en—endodermis, s—sclerenchyma, vb—vascular bundle, ep—epithelial cell, p—trichome basal cells, t—trichome stalk cells, ap—apical cell. Scale bars: 100 μm (**i**), 50 µm (**a**,**c**,**j**), 30 µm (**d**–**h**,**k**,**l**,**o**,**p**), 10 µm (**b**,**m**,**n**).

Stem: In the control preparations, the secretion contained in the secretory canals was light yellow-green. In the cross-sections of the stems, the secretory canals were shown to contain EO (Figure 4a–c) and neutral lipids (Figure 4d,e), which confirmed the production of EO by the secretory canal epithel. EO also formed droplets in stem cells adjacent to the canals (Figure 4a,c), whereas total lipids were present in the cuticle on the stem surface (Figure 4a).

The analyses showed secretory activity of both the sparse glandular trichomes (Figure 4g,h) and the basal cells (4–5 cells) of the non-glandular trichomes (Figure 4i–p) present on the surface of the stems. Glandular trichomes produced EO (Figure 4f) and neutral lipids (Figure 4g,h), whereas non-glandular trichomes produced total and acidic lipids (Figure 4i–p).

### 2.4. Analysis of the EO Composition

**Figure 5:**
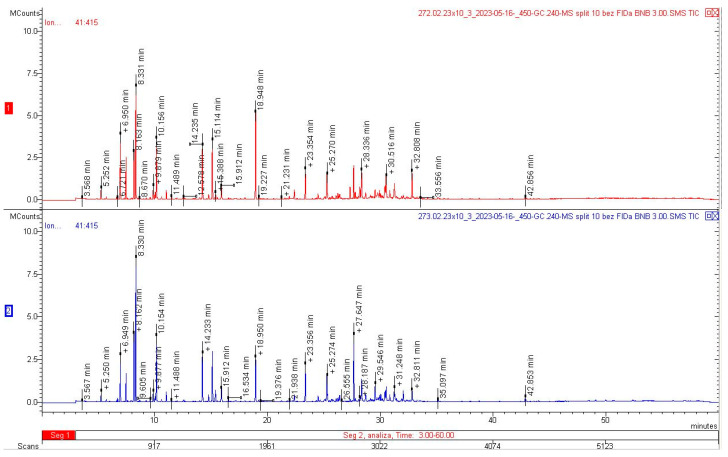
Chromatograms of EOs extracted from *Achillea millefolium* L. subsp. *millefolium* herb (**1**) and flowers (**2**).

As shown in the present study, the dried herb contained 2.4 mL/kg of EO, whereas 2.5-fold greater amounts were obtained from the flowers, i.e., on average 6.1 mL/kg. These levels accounted for 0.235% and 0.598%, respectively. The EO extracted from the herb of *Achillea millefolium* subsp. *millefolium* contained 70 substances, which accounted for 96.24 relative percentages. In turn, 68 compounds (95.04 relative percentages) were detected in the EO from the flowers (Table 4, Figure 5).

**Figure 6:**
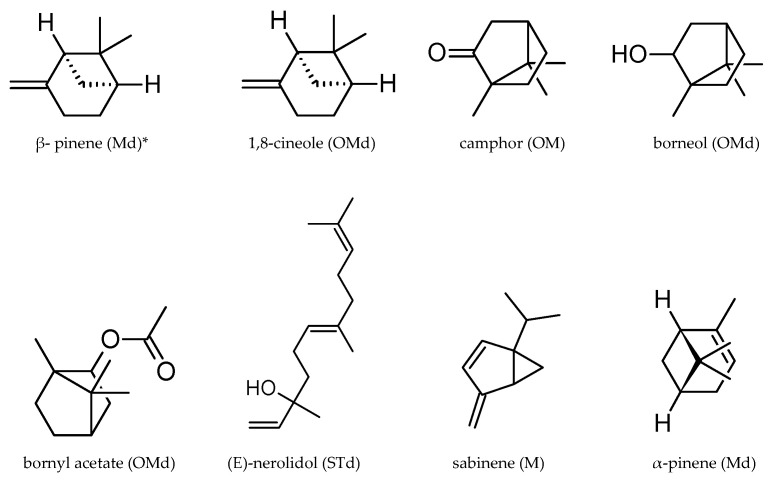
Structures of the main EO components in *Achillea millefolium* subsp. *millefolium* (≥5%). * Md—monoterpenoids; OMd—oxygenated monoterpenoids; OM—oxygenated monoterpenes; STd—sesquiterpenoids; M—monoterpenes.

β-pinene turned out to be the main component of both EOs, representing 9.90% and 12.22%, respectively, in the herb and flowers. Substantial amounts (over 5%) of 1,8 cineole (5.83% and 6.45%), camphor (5.81% and 5.28%), and borneol (6.18% and 5.25%) were detected in both EO samples. In terms of the qualitative composition, the EO extracted from the herb and flowers was similar, and there were substantial differences only in the amounts of individual components, especially bornyl acetate, accounting for 9.23% in the herb and 4.81% in the flowers, and α-pinene, whose content was 5.29% and 3.83%, respectively. In turn, the flower EO contained greater amounts of (E)-nerolidol (7.34% vs. 3.47% in the herb), sabinene (6.04% vs. 4.18% in the herb), and β-pinene (12.22 and 9.90%) (Table 4; Figure 5 and Figure 6).

**Figure 7:**
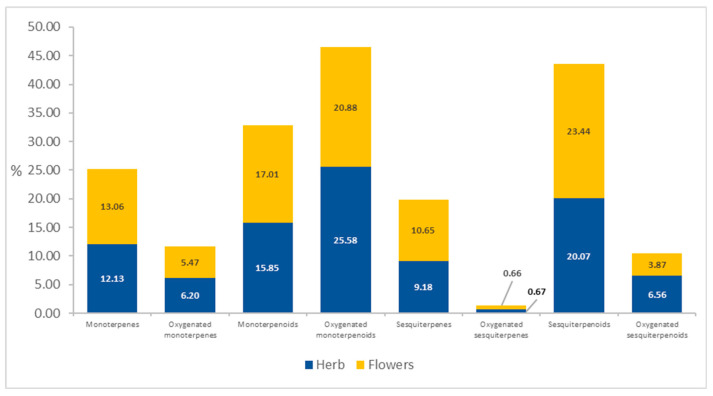
Distribution of EO compounds in *Achillea millefolium* subsp. *millefolium* flowers and herb.

The EO from the herb of *Achillea millefolium* subsp. *millefolium* had higher content of oxygenated compounds, mainly oxygenated monoterpenoids (25.58%), compared to the EO extracted from the inflorescences (20.88%). The herb also contained greater amounts of oxygenated sesquiterpenoids (6.56%) than the flowers (3.87%). Both EO samples had greater amounts of monoterpenes with derivatives than sesquiterpenes and their derivatives (Figure 7).

## 3. Discussion

### 3.1. Structure and Histochemistry

As in other Asteraceae representatives, the shoots of yarrow plants are composed of both exogenous and endogenous secretory tissues [51,52]. The exogenous secretory structures include biseriate glandular trichomes, which are typical of plants from the family Asteraceae. In the present study, the secretory trichomes in *A. millefolium* subsp. *millefolium* were located on the corollas of disc and ray flowers, receptacular bracts, and involuclar bracts. A similar distribution of trichomes on floral corollas and/or involuclar bracts in other Asteraceae representatives has been reported by Ferreira and Janick [61], Cornara et al. [62], Heinrich et al. [63], Sulborska [64,65], and Haratym et al. [53]. The glandular trichomes located on the stems, leaves, and flowers of *A. millefolium* subsp. *millefolium* were composed of three tiers of secretory cells. The present analyses revealed that the cells contained EO and substances typical of EO, i.e., neutral lipids and sesquiterpenes.

We also found these EO components in the epidermis cells of the corolla teeth and tubes of the small disc flowers. The apical zones of the adaxial surface of the teeth had conical cells (papillae), which contained EO as well. Conical cells located on flower petals frequently release scent [66]. The other functions of conical cells, which are most often found in the top region of petals, i.e., a site of direct contact with pollinators, are to increase their grip on the flower and decrease flower wettability [67,68,69].

The higher content of EO in *A. millefolium* subsp. *millefolium* flowers than in shoots (i.e., flowers, stems, and leaves) was associated with the presence of EO not only in the glandular trichomes located on the petals, receptacular, and involuclar bracts, but also in the petal epidermis cells (including papillae).

### 3.2. Quantity and Quality of EO

As indicated by the European Pharmacopoeia [18], whole or cut dried flowering tops of shoots containing at least 2 mL/kg of EO are the *Achillea millefolium* medicinal raw material. In the present study, the dried herb contained 2.4 mL/kg of EO, while the distillation yield from the inflorescences was on average 6.1 mL/kg; these values accounted for 0.235% and 0.598%, respectively. Other studies conducted in Poland reported highly diverse EO content ranging from 0.17 to 0.60% in *A. millefolium* herb originating from natural habitats [70]. Zawiślak and Nurzyńska-Wierdak [71] found that yarrow herb collected from wild plants had significantly higher amounts of EO (on average 0.5%) than herb of cultivated plants (on average 0.3%). Similarly, Benedek et al. [72] reported highly diverse levels of EO (from 0.5 to 5.88%) in commercial *Achillea* samples from Germany, Austria, France, Italy, the Netherlands, Great Britain and Bosnia-Herzegovina and in plants collected in the wild in Eastern European countries (Poland, Romania, Slovakia, Czechia, Hungary); however, the researchers did not focus on differences in the EO content between yarrow herb and flowers. In turn, Mohammed et al. [13] reported that the yield of EO from *Achillea* samples collected in Saudi Arabia ranged from 0.33% to 0.65%, with differences between the distillation yield from leaves, stems, and aerial parts of *A. millefolium*; the lowest yield was obtained from leaves. However, the authors did not specify the stage of plant development during collection or the presence and quantity of flowers in the collected aerial parts.

The EO from the inflorescences and herb of *A. millefolium* subsp. *millefolium* extracted in the present study contained the following main ingredients: β-pinene, bornyl acetate, (E)-nerolidol, 1,8-cineole, borneol, sabinene, camphor, and α-pinene. They were reported as the main components in many samples of yarrow raw material analysed in various countries: Norway (α-pinene, β-pinene, borneol, bornyl acetate, sabinene) [73], Estonia (β-pinene, 1,8-cineole, sabinene) [74], Iran (1,8-cineole, camphor) [28], and Lithuania ((E)-nerolidol, β-pinene and 1,8-cineole) [75]. However, we did not identify azulene or chamazulene, which were previously shown in many studies on *Millefolii herba* [9,28,57,76,77]. Azulenes, which are formed from proazulenes, are products of the dehydration of sesquiterpenes [78]. Likewise in the present study, various authors reported that yarrow EO did not contain chamazulene [13,79,80]. Orav et al. [56] found considerable differences in the composition of EO (19 samples) from *A. millefolium* growing in various European countries. They identified both chemotypes that were rich in chamazulene and chemotypes that did not contain this compound. In turn, Mockute and Judzentiene [81] identified four chemotypes of yarrow EO depending on the plant growth environment. Forty samples of EO were collected from plants growing in the wild in 21 localities of Lithuania; fourteen samples did not contain chamazulene, and two samples contained only traces of this component. The level of EO in 18 samples was in the range of 0.1–6.8%, and six samples contained 9.8–23.2% of EO. Based on these reports, the EOs analysed in the present study, which were characterised by the absence of chamazulene and the presence of β-pinene + 1,8-cineole, can be classified as chemotype IV in the classification proposed by Mockute and Judzentiene [81].

In the present study, the flowers of the analysed species contained higher levels of β-pinene and (E)-nerolidol than the herb. Inflorescence samples analysed by Judzentiene and Mockute [75] also contained substantially higher amounts of these compounds than leaves.

According to the requirements of the European Pharmacopoeia [18], the *Achillea millefolium* EO, which does not contain proazulenes, does not meet the requirements for use in traditional medicine. Although proazulenes are a measure of the quality of *Achillea millefolium* raw material for medical use, it should be emphasised that other EO components of this taxon also have great application-related importance. α- and β-Pinene and other dominant compounds have healing properties and are used in the cosmetic industry and for food flavouring [7,12,82,83,84,85,86].

The results of the present morpho-anatomical analyses and histochemical assays indicating the localisation of EO in the examined organs of *A. millefolium* subsp. *millefolium* correspond to the EO quantities revealed by phytochemical studies.

## 4. Materials and Methods

### 4.1. Plant Material

The materials for anatomical and phytochemical studies were *Achillea millefolium* subsp. *millefolium* L. capitula composed of ray and disc flowers (*Millefolii flos*) and upper parts of flowering shoots, i.e., herbal raw material (*Millefolii herba*). The research material was collected at the turn of July and August 2022 from natural meadows in Wola Zadybska near Lublin (the south-eastern Poland) (51°44′49″ N; 21°50′38″ E. The botanical identification of the plants was made by taxonomy specialist Professor Bożena Denisow. The collected plant specimens were also compared with authentic samples deposited in the Department of Botany and Plant Physiology, University of Life Sciences in Lublin (Poland) (no. 98).

### 4.2. Microscopic Preparation

#### 4.2.1. Light Microscopy (LM)

Ten fresh capitula, from which disc (*n* = 20) and ray (*n* = 20) flowers were randomly sampled, were selected for microscopic observations. Fresh shoots in the full flowering phase (*n* = 10), from which 1 cm long stem fragments were cut off 0.5–2 cm below the inflorescence, and leaves (*n* = 10) were used as well. The preliminary observations were performed with the use of an STM 800 stereoscopic microscope (Microlab, Shenzhen, China), and photographs were taken for documentation.

Cross-sections of the stem and leaf were made by hand, and then microscopic slides were prepared in glycerine and water (1:1). Microscopic observations were performed using a Nikon Eclipse 400 light microscope (Nikon, Tokyo, Japan). The biseriate glandular trichomes located on the surface of the ray and disc flowers, receptacular bracts, involuclar bracts, leaves, and stems were subjected to morphometric measurements. Their length and width were measured at the widest point (*n* = 30 each). The length and width of non-glandular trichomes located on the stems, leaves, receptacular bracts, and involuclar bracts were measured as well (*n* = 30 from each organ).

Semi-thin sections of fresh stems and leaves were prepared. One-centimetre-long stem fragments were taken from the middle part of the third internode (counting from the inflorescences). One-centimetre-long fragments of the leaf blade with the midrib were collected from the apical part. The plant material was fixed in 2.5% glutaraldehyde in phosphate buffer (pH 7.2; 0.1 M) at 4 °C for 12 h. In the next step, the samples were washed three times in phosphate buffer, dehydrated in an ethanol series, and embedded in LR white resin (LR white acrylic resin, medium grade, Sigma-Aldrich, Saint-Louis, MO, USA). Semi-thin sections (0.7–0.9 μm) were cut transversally using a Reichert Ultracut S ultramicrotome (C. Reichert Optische Werke AG, Vienne, Austria) and a glass knife. For general histology, the sections were stained with a 1% (*w*/*v*) aqueous methylene blue-azure II solution [87]. The sections were examined using an Olympus CX 23 light microscope (Olympus, Tokyo, Japan) equipped with an Olympus EP50 digital camera (Olympus, Tokyo, Japan) and EPview software ver. 1.2.

#### 4.2.2. Scanning Electron Microscopy (SEM)

The surfaces of the stems, leaves, and flowers were observed using SEM. Stem and leaf fragments 0.5 cm long (*n* = 10) were fixed in a 2.5% glutaraldehyde solution in 0.1 M phosphate buffer (pH 7.0) at room temperature for 2 h and then washed in phosphate buffer four times at 20-min intervals. The fixed plant material was dehydrated in graded ethanol series and immersed in absolute ethanol (POCH, Gliwice, Poland) three times for 30 min. Next, the flower samples were critical-point dried in liquid CO_2_ using a K 850 Critical Point Dryer and sputter-coated with gold (20 mm thickness) using a K 550X device (Emitech, Ashford, UK). The observations were made with the use of a Tescan Vega II LMU scanning electron microscope (Tescan, Brno, Czech Republic) at an accelerating voltage of 30 kV. Images were taken using a BSE detector and saved using Vega software ver. 3.5.19. 

### 4.3. Histochemical Assays

Disc flowers, receptacular bracts (*n* = 20 each), and hand-made cross-sections of the stems (0.5–2 cm below the inflorescence; *n* = 10) were subjected to histochemical analyses (*n* = 6) to identify lipophilic compounds present in these organs and determine their location. Sudan IV [88,89] and Neutral red [90,91] staining was employed to identify total lipids and EO, Nile blue was used to detect acidic and neutral lipids [92,93], and sesquiterpenes were detected using concentrated sulphuric acids [94,95]. The flowers and stem sections were also used to make control aqueous glycerol preparations (1:1). All preparations were observed under an Olympus CX23 light microscope (Olympus, Tokyo, Japan) equipped with a digital camera Olympus EP50 (Olympus, Tokyo, Japan) and EPview software ver. 1.2.

### 4.4. Phytochemical Analysis

**Figure 8:**
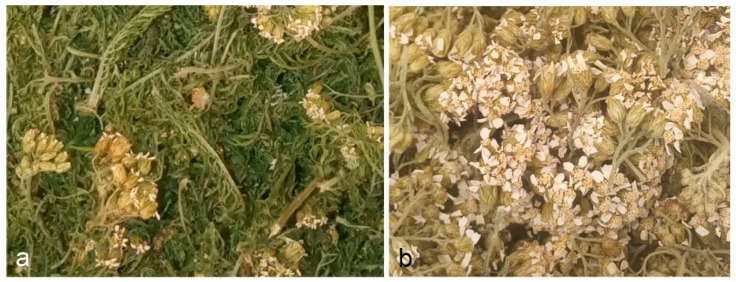
Samples of *Achillea millefolium* subsp. *millefolium* herb (**a**) and flowers (**b**) intended for distillation of EOs.

Fully blooming yarrow shoots were cut at a height of approximately 20 cm above the ground. Immediately after harvesting, the material was dried in a dryer with forced air circulation at a temperature of 35 °C. The samples were dried for 2 h to achieve a constant humidity level of 12%. The dried material was mixed and homogenised to obtain herb containing flowers, leaves, and thin fragments of stems, whereas fully blooming inflorescences were separated (Figure 8). In the study, we used 500 g of dry herb material and 300 g of floral material. Fifty-gram samples of the plant material were hydrodistilled in a Deryng apparatus for 3 h. The distillation process was repeated several times to obtain at least 1 mL of EO from each sample type. We obtained 1.8 mL of floral EO and 1.2 mL of herb EO from the collected material. The content of EO in the raw material was expressed in mL/kg. The EO samples were diluted 1000-fold in hexane. The analysis of the EO composition was carried out using a gas chromatograph with a 450-GC + 240-MS Varian mass detector at the Central Research Laboratory of the University of Life Sciences in Lublin.

The following conditions were used: sample dose: 1 µL, dispenser: 250 °C, split 1:10, carrier gas: helium, constant flow 1.0 mL/min, column: VF-5ms (DB-5 equivalent), column oven: 50 °C held for 1 min, ramp to 240 °C at a rate of 4 °C/min, 240 °C held for 10 min, total analysis time: 60 min, detector: mass spectrometer with an ion trap: electron ionisation EI, full scanning: in the range of 41–415 *m*/*z*, scanning speed: 0.8 s/scan. The compounds were identified based on mass spectra and “Identification of Essential Oil Components by Gas Chromatography/Mass Spectrometry, ed. 4.1; Dr. Robert P. Adams Professor Biology Department Baylor University” and the NIST Mass Spectra Library.

Kovats retention indices were determined on the VF-5ms column (equivalent to DB-5) using a series of n-alkanes (C7-C40 Saturated Alkanes Standard, Product no.: 4 9452-U Supelco) as in Van Den Dool and Kratz [96].

## 5. Conclusions

The stems, leaves, and flowers of *Achillea millefolium* subsp. *millefolium*, which are medicinal and cosmetic raw material, were found to have exo- and endogenous secretory structures responsible for the production and accumulation of EO components. The presence of EO was detected in glandular trichomes, papillae, and secretory canals. In turn, sesquiterpenes, which are important components of EO as well, were detected not only in glandular trichomes but also in disc flower epidermis. The analysis of the distillation yield from the herb samples (2.4 mL/kg) and flowers (6.1 mL/kg) of *A. millefolium* subsp. *millefolium* showed that the flowers contained substantially higher amounts of EO than the other parts of the shoot. The composition of the EO extracted from both samples was largely similar, with higher content of monoterpenes and their derivatives than sesquiterpenes and their derivatives. In turn, the herb EO exhibited a higher number of oxygenated monoterpenes and oxygenated sesquiterpenoids than the EO obtained from the flowers. The analysed *A. millefolium* subsp. *millefolium* plants represented the azulene-free type. The EOs extracted from both the herb and flowers represent the β-pinene + 1,8-cineole chemotype. It can be expected that, given the biological and medicinal activity of the dominant EO components, the analysed yarrow chemotype can be used in the pharmaceutical and cosmetic industries and for food flavouring. As shown in this study, the presence of various secretory structures in the flower epidermis (more numerous glandular trichomes and papillae) contributed to the higher amounts of EO produced by the flowers than by the herb.

## Figures and Tables

**Table 1:** Morphometric traits of glandular trichomes located on the organs of *Achillea millefolium* subsp. *millefolium*.

Plant Organ	Height of Trichome (μm)		Width of Trichome (μm)	
	Min–Mix	Mean ± SD	Min–Max	Mean ± SD
Stem	38.55–48.83	43.46 ± 5.56	30.84–64.25	42.28 ± 15.67
Leaf	51.40–53.97	52.69 ± 1.10	35.98–38.55	37.70 ± 0.89
Disc flower	64.25–82.10	71.32 ± 10.20	84.81–107.94	98.51 ± 15.21
Ray flower	56.54–82.24	69.39 ± 9.84	77.10–92.52	83.70 ± 8.65
Receptacular bract	64.25–79.67	71.96 ± 7.45	77.50–95.09	86.10 ± 8.76

**Table 2:** Morphometric traits of non-glandular trichomes in *Achillea millefolium* subsp. *millefolium*.

Plant Organ	Length of Trichome (μm)		Width of Trichome (μm)	
	Min–Max	Mean ± SD	Min–Max	Mean ± SD
Stem	765.6–2705.12	1621.17 ± 759.89	14.14–46.26	27.72 ± 14.33
Leaf	459.45–2807.75	1410.0 ± 896.21	16.30–34.28	24.66 ± 10.87
Receptacular bract	256.68–342.24	299.46 ± 38.90	15.42–17.99	16.91 ± 1.48
Involuclar bract	59.11–95.09	84.24 ± 9.85	12.85–17.99	16.0 ± 1.37

**Table 3:** Lipophilic compounds identified in trichomes and canals of *Achillea millefolium* subsp. *millefolium* by histochemical assays.

Metabolite	Reagent	Color	Ray Flowers		Inflorescence Stem	
			Glandular Trichomes	Non-Glandular Trichomes	Glandular Trichomes	Secretory Canals
Total lipids/ Essential oil	Sudan IV	orange	+	+	+	+
	Neutral red	red	+	+	+	+
Acidic lipids	Nile blue	blue	+	+	−	−
Neutral lipids	Nile blue	pink, violet	+	−	+	+
Sesquiterpenes	Conc. sulphuric acid	yellow	+	−	−	−

+ positive, − negative.

**Table 4:** Chemical composition of EOs extracted from *Achillea millefolium* subsp. *millefolium* herb and flowers.

No	Compound	Class *	Retention Time (min.)	Retention Index Acc. to Kovats	Herb %	Flowers %
**1**	xylene	M	5.25	869	0.87	0.82
**2**	tricyclene	M	6.63	922	0.17	0.13
**3**	α-thujene	M	6.72	925	0.20	0.25
**4**	α-pinene	Md	6.95	933	5.29	3.83
**5**	camphene	M	7.46	949	3.20	2.29
**6**	benzaldehyde	OM	7.92	964	0.09	0.12
**7**	sabinene	M	8.16	973	4.18	6.04
**8**	β-pinene	Md	8.33	978	9.90	12.22
**9**	myrcene	M	8.67	989	0.16	0.14
**10**	α-phellandrene	M	9.25	1007	0.07	0.10
**11**	α-terpinene	M	9.60	1017	0.15	0.29
**12**	p-cymene	M	9.88	1025	1.29	1.06
**13**	limonene	M	10.03	1030	0.60	0.42
**14**	β-phellandrene	M	10.08	1031	0.11	0.19
**15**	1.8-cineole	OMd	10.16	1033	5.83	6.45
**16**	(Z)-β-ocimene	Md	10.24	1036	0.12	0.13
**17**	(E)-β-ocimene	M	10.60	1046	0.20	0.23
**18**	γ-terpinene	M	11.03	1059	0.83	0.96
**19**	cis-sabinene hydrate	OMd	11.49	1072	0.33	0.21
**20**	terpinolene	M	11.98	1086	0.10	0.14
**21**	linalool	OMd	12.51	1101	0.08	0.08
**22**	trans-sabinene hydrate	OMd	12.58	1103	0.24	0.22
**23**	cis-p menth-2-en-1-ol	OM	13.40	1127	0.05	0.07
**24**	trans-pinocarveol	OMd	14.00	1144	0.24	0.22
**25**	camphor	OM	14.23	1151	5.81	5.28
**26**	cis-chrysanthenol	Md	14.78	1166	0.45	0.72
**27**	δ-terpineol	OMd	15.04	1173	0.10	0.09
**28**	borneol	OMd	15.12	1176	6.18	5.25
**29**	terpinen-4-ol	OMd	15.39	1183	0.80	1.23
**30**	α-terpineol	OMd	15.91	1198	1.66	1.78
**31**	trans-carveol	Md	16.75	1222	0.09	0.10
**32**	cis-chrysanthenyl acetate	OMd	18.01	1259	0.20	0.14
**33**	bornyl acetate	OMd	18.95	1287	9.23	4.81
**34**	thymol	OMd	19.23	1295	0.32	0.22
**35**	piperitenone	OM	20.82	1343	0.13	-
**36**	eugenol	OMd	21.23	1356	0.37	0.18
**37**	piperitenone oxide	OM	21.54	1365	0.12	-
**38**	α-copaene	ST	21.94	1377	0.29	0.24
**39**	β-elemene	ST	22.38	1391	0.88	0.66
**40**	(E)-caryophyllene	ST	23.35	1422	3.38	3.95
**41**	α-humulene	ST	24.48	1458	0.58	0.69
**42**	β-chamigrene	ST	25.03	1475	0.25	0.25
**43**	γ-curcumene	ST	25.14	1479	0.22	0.36
**44**	germacrene D	ST	25.27	1483	2.91	3.33
**45**	α-zingiberene	ST	25.69	1497	0.08	0.22
**46**	epi-cubebol	STd	25.73	1498	0.29	0.25
**47**	α-cuprenene	ST	25.85	1502	0.06	0.16
**48**	sesquicineole	STd	26.25	1515	0.55	0.47
**49**	cubebol	STd	26.34	1518	0.28	0.17
**50**	δ-cadinene	ST	26.40	1521	0.53	0.79
**51**	β-sesquiphellandrene	STd	26.55	1526	0.12	0.22
**52**	elemol	STd	27.32	1552	1.26	0.26
**53**	(E)-nerolidol	STd	27.65	1563	3.47	7.34
**54**	spathulenol	STd	28.19	1581	1.51	0.91
**55**	caryophyllene oxide	OSTd	28.34	1586	3.54	2.70
**56**	viridiflorol	STd	28.70	1598	0.92	1.14
**57**	humulene epoxide II	OST	29.15	1614	0.35	0.33
**58**	eremoligenol	STd	29.55	1628	0.97	2.13
**59**	γ-eudesmol	STd	29.77	1636	0.55	0.62
**60**	caryophylla-4(14).8(15)-dien-5-α-ol	STd	29.93	1642	0.83	0.65
**61**	epi-α-cadinol	STd	30.04	1646	0.41	0.86
**62**	aromadendrene epoxide	OST	30.19	1651	0.32	0.33
**63**	himachalol	STd	30.42	1660	1.98	1.38
**64**	neo-intermedeol	STd	30.52	1663	2.76	1.62
**65**	14-hydroxy-9-epi-(E)-caryophyllene	STd	30.84	1675	0.90	0.77
**66**	germacra-4(15).5.10(14)-trien-1α-ol	STd	31.25	1689	2.29	2.66
**67**	amorpha-4.9-dien-2-ol	STd	31.42	1695	0.24	0.46
**68**	shyobunol	STd	31.53	1699	0.16	0.14
**69**	(2Z,6E)-farnesol	STd	32.03	1718	0.58	1.39
**70**	6S,7R-bisabolone	OSTd	32.81	1747	3.02	1.17
	Total %				96.24	95.04

* M—monoterpenes; OM—oxygenated monoterpenes; Md—monoterpenoids; OMd—oxygenated monoterpenoids; ST—sesquiterpenes; OST—oxygenated sesquiterpenes; STd—sesquiterpenoids; OSTd—oxygenated sesquiterpenoids.

## Data Availability

The datasets used and/or analysed during the current study are available from the corresponding author on reasonable request.
